# 

**Published:** 1910-12-31

**Authors:** 


### CONTENTS.

| | PAGE |
|---|---|
| **Style in Medical Authorship** | 397 |
| **The Medical Year** | 398 |
| **Annotations** - The Houndsditch Murders—Public Health and Local Administration—The late Professor Huchard | 399 |
| **Medical Opinion and Movement** | 400 |
| **Hospital Clinics**— Some Untoward Consequences of Phimosis and of Circumcision. By Geo. H. Edington, M.D., F.R.F.P.S.G. | 403 |
| **Special Article**— The Evolution of Joint Surgery | 405 |
| **Anæsthetics**— The Anæsthetist's Outfit | 4C6 |
| **Medicine**— Grain Itch | 407 |
| Vincent's Angina | 407 |
| **Diseases of Children**— Epidemic Parotitis | 409 |
| **Practical Notes on Diagnosis and Treatment** | 410 |
| **News and Events of the Week** | 411 |
| **Editor's Letter-Box**— "The West London Appointment"—"An Advance Announcement"—The Height of Antisepsis | 412 |
| **Parliament and Publications** | 412 |
| **Appointments and Resignations** | 412 |
| **The Week's Work**— The West London Hospital Appointment—Christmas and the Hospitals—Christmas Souvenirs—The Past Year—The Death List—Questions of the Day—This Week's Drug Market | 413 |
| **Special Institutional Articles**— Royal Southern Hospital, Liverpool | 415 |
| German Hospital, Dalston | 417 |
| The Westminster Hospital Medical School | 418 |
| Hospital Relief in Holland | 419 |
| **Institutional Notes and News** | 421 |
| **The Question Box** | 421 |
| **Gifts and Bequests** | 422 |
| **Jottings** | 424 |
| **The Hospital World** | 425 |
| **New Appliances and Things Medical** | 426 |

